# The Pumpkin or the Tiger? Michael Polanyi, Frederick Soddy, and Anticipating Emerging Technologies

**DOI:** 10.1007/s11024-012-9204-8

**Published:** 2012-09-06

**Authors:** David H. Guston

**Affiliations:** Politics and Global Studies, Consortium for Science, Policy and Outcomes, Center for Nanotechnology in Society, Arizona State University, 1120 S. Cady Mall, Tempe, AZ 85287 USA

**Keywords:** Michael Polanyi, Frederick Soddy, Anticipatory governance, Emerging technologies, Atomic bomb, Leo Szilard, H.G. Wells

## Abstract

Imagine putting together a jigsaw puzzle that works like the board game in the movie “Jumanji”: When you finish, whatever the puzzle portrays becomes real. The children playing “Jumanji” learn to prepare for the reality that emerges from the next throw of the dice. But how would this work for the puzzle of scientific research? How do you prepare for unlocking the secrets of the atom, or assembling from the bottom-up nanotechnologies with unforeseen properties – especially when completion of such puzzles lies decades after the first scattered pieces are tentatively assembled? In the inaugural issue of this journal, Michael Polanyi argued that because the progress of science is unpredictable, society must only move forward with solving the puzzle until the picture completes itself. Decades earlier, Frederick Soddy argued that once the potential for danger reveals itself, one must reorient the whole of one’s work to avoid it. While both scientists stake out extreme positions, Soddy’s approach – together with the action taken by the like-minded Leo Szilard – provides a foundation for the anticipatory governance of emerging technologies. This paper narrates the intertwining stories of Polanyi, Soddy and Szilard, revealing how anticipation influenced governance in the case of atomic weapons and how Polanyi’s claim in “The Republic of Science” of an unpredictable and hence ungovernable science is faulty on multiple levels.

## The Puzzle

Imagine you’re putting together a jigsaw puzzle. This puzzle, however, works a bit like the board game in the movie “Jumanji”: When you finish, whatever the puzzle portrays becomes real.

The children playing “Jumanji” quickly learn to prepare for the reality that emerges from the next throw of the dice. But how would this work for the puzzle of scientific research? How do you prepare for unlocking the secrets of the atom, or piecing together the genome of a bacterium new to evolution, or assembling from the bottom-up nanotechnologies with unforeseen properties, or engineering the climate in an attempt to ward off catastrophic global warming – especially when completion of such puzzles lies decades after the first scattered pieces are tentatively assembled?

One response is that preparation is vastly more complicated than it is in children’s movies. In the words of the eminent chemist and philosopher of science Michael Polanyi (2000 [1962]: 9), such preparation is “impossible and nonsensical” because science advances “only by essentially unpredictable steps, pursuing problems of its own,” and the practical consequences of “these advances will be incidental and hence doubly unpredictable” (Polanyi 2000 [1962]: 10).^1^ That is, because you can’t predict what the puzzle portrays, you just need to move forward until the picture completes itself.

Not all scientists would agree, including another eminent chemist, Frederick Soddy, who believed that it is the “duty” of the scientists doing the puzzling “to spend our lives and brains thinking” such things “out for ourselves” (cited in Sclove 1989: 179). That is, once the potential for danger reveals itself, you must reorient the whole of your work to avoid it.

Polanyi’s response commands the allegiance of most contemporary researchers. It has informed the public policies that support scientific research and has surely contributed to their enviable successes. It also rushed us blindly, in Polanyi’s day, into atomic energy, as it now hurtles us into synthetic biology, nanotechnology and geoengineering.

Soddy’s alternative is attractive, but demanding. Following his own advice, he all but wrote himself out of the scientific community despite his Nobel Prize. And surely Polanyi is right to say that none of this can be predicted – in the technical sense. Only hindsight may say in fact if synthetic biology will bring bounty or plague, if nanotechnologies will be more benign or malign than industrial chemistry, or if geoengineering will soften the blow of climate change or make it graver. But must the rest of us – who expect so much good from science and yet remain apprehensive of its directions – simply resign ourselves to whatever researchers, like the Fates, might have in store?

As scientists puzzle out these pictures of nature, say, warm with orange and deep with black, how do we best respond to the realization that the picture could be a pumpkin…or a tiger?

## Polanyi and Prediction

In January 1945, Michael Polanyi crowded in a BBC radio booth with his friend Bertrand Russell, three other guests, and the host of the Brains Trust. Russell, with wild tufts of white topping his bulbous head, perhaps chewing his pipe, was something of an aged Sherlock Holmes. Polanyi, thin-lipped and precise but with warm, dark eyes, could have been his Watson. A large, black, lozenge-shaped microphone dominated the bridge-sized table and carried their answers to callers’ questions to England’s largest radio audience of the day.^2^

**Fig. 1:**
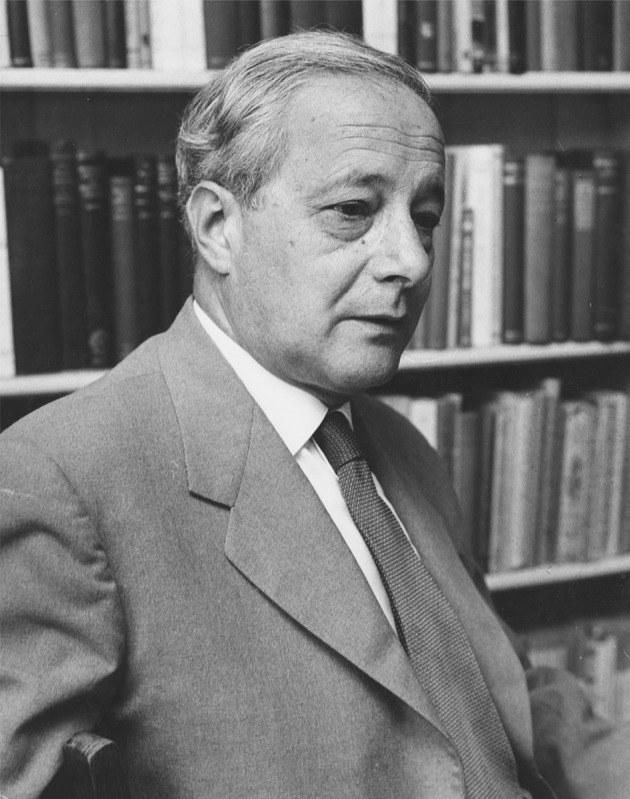
Professor Michael Polanyi, F.R.S. From the Michael Polanyi Papers, Special Collections Research Center, University of Chicago, Box 45, folder 3

Polanyi recounts a segment of this broadcast in his 1962 essay, “The republic of science,” published in the first issue of the journal *Minerva* under the heavy, green editorial pen of sociologist Edward Shils. The two formed a bond, beginning right after World War II and evident three decades later in Shils’ remembrance of Polanyi in *Minerva*: “The noble elevation of his bearing, his eloquence of speech, his compassion and his clarity of conviction were of a piece with his devotion to the discovery and possession of truth and his conception of it as one of the first obligations of a good society” (Shils 1976: 3) (Fig. 1).

In his essay, Polanyi draws from the radio booth an anecdote that illustrates the unpredictability of science. A caller asked “about the possible technical uses of Einstein’s theory of relativity, and [none] of us could think of any….But actually,” Polanyi (2000 [1962]: 9–10) admits, “the technical application of relativity…was to be revealed within a few months by the explosion of the first atomic bomb.”

Polanyi argues that because science is unpredictable, then its subsequent technical and social outcomes are even more so. He weaves an intricate analogy between the conduct of science and the play of the economic market, both of which exemplify how individuals can maximize socially beneficial outcomes by pursuing their own interests and adjusting, mutually but independently, to the interests of others. The same “invisible hand” that guides the market guides science.

While he allows that “Russell and I should have done better in foreseeing these applications of relativity in January 1945,” he extends their own incapacity back a half century by also arguing that “Einstein could not possibly take these future consequences into account when he started on the problem which led to the discovery of relativity” because “another dozen or more discoveries had yet to be made before relativity could be combined with them to yield the technical progress which opened the atomic age” (Polanyi 2000 [1962]: 10).

Polanyi concludes that the corresponding march of knowledge and ignorance from Einstein’s patent office to the BBC radio booth is critical to the progress of science. He memorably declares that “[a]ny attempt at guiding scientific research towards a purpose other than its own is an attempt to deflect it from the advancement of science….You can kill or mutilate the advance of science, you cannot shape it” (Polanyi 2000 [1962]:10).

## Soddy and Responsibility

**Fig. 2:**
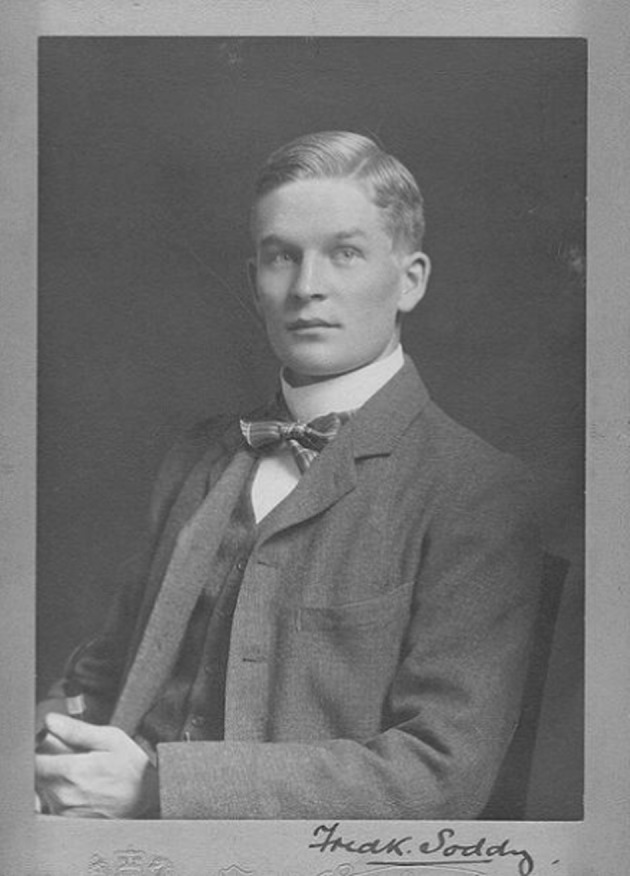
Frederick Soddy, about 1900–1903. By the kind permission of the Frederick Soddy Trust

Soddy brought to his chemistry the rugged looks, according to one commemorator, “of a Norseman” and the dynamic tension of an imaginary Michaelangelo entitled “Will Held in Chains by Reason” (Glasberg 1958). He appeared, in vigor and earnestness as well as in experimental skill, like a top baseball player of his era – the Honus Wagner of radioactivity. He achieved his eminence through some of those discoveries Polanyi thought clouded the vision between Einstein and the bomb: Soddy contributed fully to the research on the transmutation of the elements for which his senior partner, Ernest Rutherford, won the 1908 Nobel Prize in Chemistry. In 1903, he collaborated with William Ramsay to isolate helium from radium emissions, and Ramsay won the Nobel Prize the next year. Soddy finally won his own laurels in 1921 for the theory of isotopes (Fig. 2).

He also anticipated what Polanyi and Russell could not predict.

Early on, Soddy and Rutherford began to clarify the nature of transmutation as radioactive decay and of the quantity of energy involved in this process, which they understood to be millions of times more powerful than known chemical reactions and explosives. Soddy suspected that transmutation and the release of the atom’s internal energy were intimately linked. He seems to have been the first, in 1903, to use the term “atomic energy” (Davies 1992: 359) and with Rutherford even imagined that such a process was crucial in the creation of energy by stars.

Soddy quickly became engaged in popular writing and speaking about the new discoveries and his vision for the uses to which they would be put. Ultimately, he believed, the energy from the transmutation of the atom would provide power to drive utopian transformations of the globe – even though he had no idea of what mechanism would induce transmutation to liberate this atomic energy, or what mechanism would control it.

Like many in Europe, Soddy’s optimism about the uses to which scientific knowledge might be put itself transmuted with the experience of The Great War. While technically challenged by the war-time research agenda imposed on him at the University of Aberdeen, he lamented the destructive outcomes – particularly pressing the Haber process to fix nitrogen for fertilizers into service to instead make high explosives.

A reading of H.G. Wells’ 1914 novel, *The World Set Free*, which was dedicated to Soddy’s earlier, popular account of *The Interpretation of Radium*, reinforced the lesson of the Haber process that governments would use any technical resource to secure military advantage. By 1915, according to an account by his nephew, Soddy had “recoiled from his glance into the abyss of nuclear warfare…[and] the career of a great scientist appears to have gone progressively sour on him” (quoted in Davies 1992: 357).

## A Natural Experiment

So we have two stories about foreseeing the atomic bomb – almost a natural experiment in which circumstances hand us a comparison so close that it might have been controlled in a laboratory: One eminent chemist who hit a bull’s eye from three decades out, and another that missed the mark entirely from just a few months away.

The point is not to play “gotcha!” with Polanyi, as the contemporary press plays with politicians, for his hyperopia. From Greek mythology to global climate change, prediction is a messy and often misguided business (e.g., Sarewitz et al. 2000). There is no straight-line extrapolation from Einstein’s articulation that the speed of light is a constant that describes the relationship between mass and energy to the ability, willingness, and commitment to design, test, deploy and drop on a human population an atomic bomb.

Nevertheless, in January 1945, as Polanyi and Russell sat dumbfounded in the BBC radio booth, others were building on the work of thousands of scientists, engineers, and laborers to finalize the designs for Fat Man and Little Boy. In order for the Manhattan Project to have assembled such an effort, someone must have envisioned the completed puzzle that Polanyi and Russell did not. This someone was Leo Szilard, like Polanyi a Hungarian-Jewish émigré, who had his principal insight in 1933 and, in 1939, contacted Einstein to write the letter about the possibility of a bomb to President Franklin Roosevelt.

Soddy’s foresight does not prove that prediction is possible. He deserves his due, but not because, as scientists like to say, “the truth will out.” The truth that willed out might have been another way, and the Manhattan scientists, for example, might have made the same poor choices that misdirected and dispirited their German counterparts. Rather, Soddy deserves his due for engaging the public about serious issues even as he was grappling with them as a scientist, and for envisioning a variety of outcomes of his research even from the midst of their unpredictability.

Indeed, what distinguishes Soddy and Polanyi is precisely their conception of the relationship between how much of the puzzle is done and what responsibility we have to act on a partial image.^3^ Polanyi maintains that the ability to discuss the future practical uses of a discovery must be grounded in the most concrete and complete technical understanding – as if the outcome must be a necessary conclusion of that technical understanding. Yet, we cannot know that conclusion until it is upon us because any step between a premature prediction and a *fait accompli* may turn in a new and unforeseen direction.

This belief that we would have to do the impossible (predict scientific outcomes) in order to do the desirable (steer science toward good social outcomes) allows Polanyi to take advantage of the unsound logic at the heart of his radio booth anecdote: Because he and Russell are smart, and they didn’t think of the bomb, then it cannot be thought of. Polanyi abdicates responsibility not only on his and Einstein’s behalf, but also on behalf of all scientists in between. Even Shils (1976:4) in eulogizing his friend admitted that he would place “greater weight than Michael Polanyi on the obligations which scientists and scholars and scientific and academic institutions have for the well-being of their societies.”

Soddy’s anticipation rebuts Polanyi’s claim about Einstein and the intervening scientists. Yet having spent the remainder of his life “thinking this thing out,” Soddy tragically failed on two counts: He was unable to convince a broader public that atomic weapons were a foreseeable and dangerous consequence of the physics being pursued between the world wars, and he was unable to have even the technical aspects of his argument register in the minds of scientists like Polanyi.

## Did the Dog Bark?

**Fig. 3:**
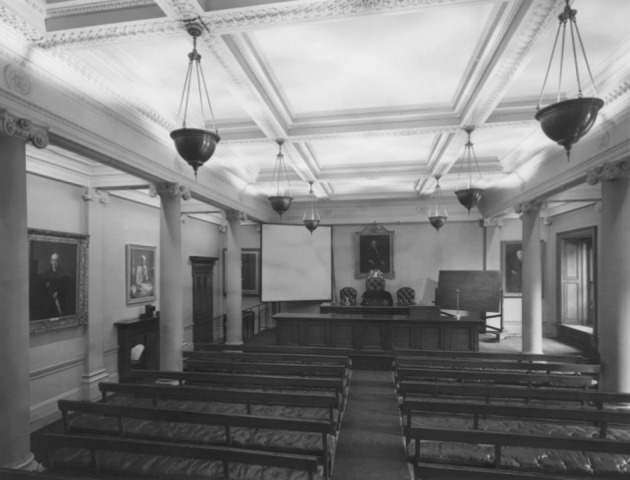
The meeting room at the Royal Society’s Burlington House, circa 1939. Copyright and permission The Royal Society

Soddy sat, no doubt uncomfortably, listening to his mentor address the fellows of the Royal Society of London in the meeting room of its stolid, inter-war home in Burlington House. A portrait of Newton peered down on the proceedings. Broad, deep-eyed and mustached, Ernest Rutherford had been elected a fellow shortly after his collaboration with Soddy. Knighted and granted a barony for his scientific contributions, he served for five years as president of this, the world’s oldest continuing scientific society. Within a few years Rutherford would be dead, but a portrait hung in the front of this same meeting room, not five meters from Newton’s left hand, would mark his own scientific apotheosis.^4^ This night, Rutherford led a “Discussion on Heavy Hydrogen.” He detailed advances in isotope research that had been leaving his now estranged protégé behind (Rutherford et al. 1934) (Fig. 3).

Soddy responded forcefully to Rutherford that evening – as he had in a debate with Rutherford nearly forty years earlier that had brought his willful intellect to Rutherford’s attention. Immediately after Soddy at the lectern, and perhaps seated next to him at the dais throughout the discussion meeting, was Michael Polanyi, recently arrived at the University of Manchester after fleeing the Nazi’s Nuremberg laws. It was clearly not because Soddy was unknown to Polanyi that his discussions of an atomic bomb failed to impress him.

Knowing Soddy in this narrow context might not mean being aware of his efforts to publicize the technical consequences of their common atomic research. But it was well-known by the time of the hydrogen discussion that Soddy’s scientific career was in terminal decline, having been “diverted to economic, social and political theories which gained no general acceptance, and to unusual mathematical and mechanical problems,” as his official Nobel biography put it (N.A. 1966). Polanyi’s own research on heavy hydrogen had helped put the particulars of Soddy’s Nobel theory of isotopes on the defensive.

Prior to his Brains Trust performance, Polanyi knew about Soddy’s politics as well. Just months after the publication of the hydrogen discussion, Soddy circulated a letter to fellows of the Royal Society seeking support for democratic reforms of the antiquated institution, leading to the “revolt of 1935” (see Hughes 2010). That year, Soddy (1935) also prefaced a collection of essays entitled *The Frustration of Science*. This volume elaborated the thinking of a group of British scientists known as the “scientific humanists” for their critique of the ideology of a pure science that operated in a way detached from the needs of the broader society.

Polanyi had witnessed the political destruction of the scientific community in his native Hungary, only to be surprised by a more thorough cleansing in his adopted Germany. He had also personally examined the fate of state-dominated science and economics in Soviet Russia. To his eyes, the scientific humanists posed a threat to scientific autonomy, and in 1940 he founded the Society for Freedom in Science to counter them.^5^ It would be devastating to Polanyi’s vision of a free science if the scientific humanist Soddy, decades earlier, had been able to predict the atomic bomb.

## *The World Set Free*

**Fig. 4:**
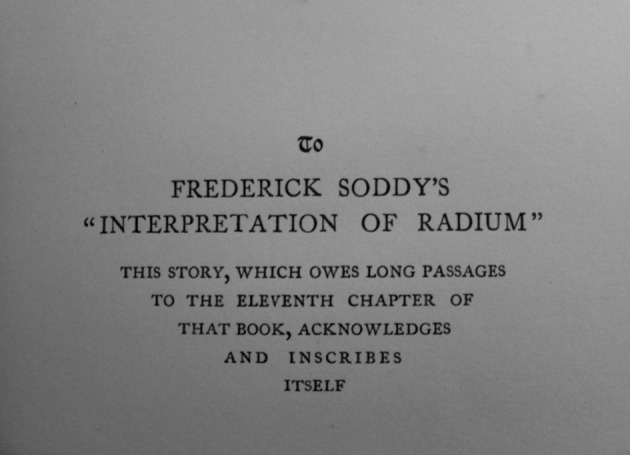
Dedication page of the first edition of *The World Set Free*. Photograph by the author

H.G. Wells dedicated his 1914 novel, which was more the rage among physical scientists than among the general public, to Frederick Soddy. While some reviewers found it compelling, the reviewer in the *New York Times* completed “it with a reluctant feeling that it is neither flesh nor fowl.” Some reviewers praised its founding “solidly on the possibilities, remote as these may now seem, of scientific developments along established lines”; others denounced it as “a charlatan piece of work, palpably and inexcusably unreal” (Fig. 4). 6

Polanyi was likely in the latter camp. As he recalled in an interview given to Thomas Kuhn in 1962, “I read H.G. Wells as a youngster and things like that. He said there would be a great war. I thought that I would make my discoveries in science before the great war and afterwards I would go on and do something in philosophy of science” (AIP 1962). While this remark was perhaps the most prescient he would make, Polanyi drew no insight into either an atomic future or the nature of conflict from Wells.

Soddy’s reading of Wells, however, initiated a radicalization that the Great War completed. In his inaugural lecture to his chemistry students at Aberdeen in October 1914, Soddy expressed only some of the ambivalence of other reviewers of *The World Set Free*. As recounted by his biographer Linda Merricks (1996), Soddy described the great human purposes to which energy from within the atom could be put, if only it could be released in a controlled fashion. He also warned his students that research “could be used for evil as well as good”; concluding his lecture, Soddy commended the “fanciful but perhaps after all not so fanciful” portrayal of atomic energy and atomic warfare provided by “Mr. Wells in his greatest novel” (Merricks 1996: 67).

Another avid reader, and indeed personal acquaintance, of H.G. Wells was Leo Szilard. Also an acquaintance of Shils (1964: 35), Szilard had “the face of a benign, sad, gentle, mischievous cherub….He had sparkling eyes, a beautiful melancholic twinkle of a smile, and spoke in a low musical voice.” He did not read *The World Set Free* until 1932 and was, as Richard Rhodes (1995 [1986]) recounts, unprepared for Wells because of the consequences for himself and his family of the mounting turmoil in Germany. Szilard had kept his suitcases packed – it’s not paranoia if they really are after you – as the prelude to the Nazi’s exterminationist approach mounted. He belittled Polanyi’s credulity under the same circumstances for his friend “thought that civilized Germans would not stand for anything really rough happening.”

Szilard’s thoughts returned to Wells later in 1933 in London, where he read an account of a speech by Rutherford describing the hope for “the liberation of atomic energy on industrial scale” as “moonshine.” In a much-cited but under-appreciated anticipation, Szilard describes his consequent brainstorm of a mechanism for the liberation of atomic energy and his motive for protecting it: “This was the first time, I think, that the concept of critical mass was developed and that a chain reaction was seriously discussed. Knowing what this would mean – and I knew it because I had read H.G. Wells – I did not want this…to become public.”

**Fig. 5:**
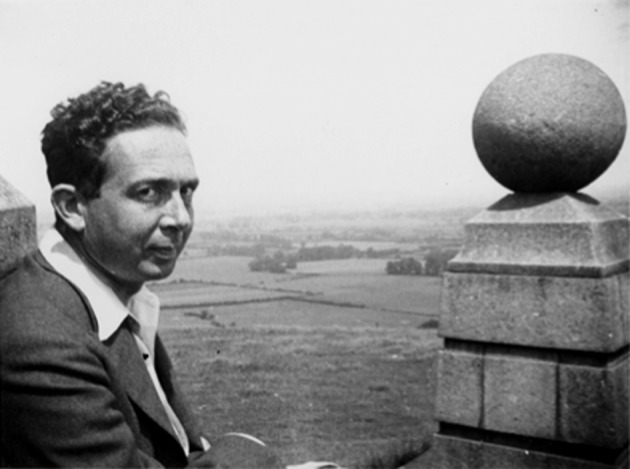
Leo Szilard in England, 1936. Photography by his wife, Gertrude Weiss Szilard. Courtesy the Leo Szilard papers. MSS 32. Mandeville Special Collections Library, UC San Diego, Box 100, Folder 15

Still, Szilard had only theorized the nuclear chain reaction and needed to investigate it. He sought funds to support new research from Chaim Weizmann, who sought advice from a fellow chemist – Michael Polanyi. While Weizmann never responded, Szilard talked further with Polanyi who – after Nuremberg – finally recognized what roughness the German people would not just stand for but embrace and fled to England. Now heading the chemistry department at the University of Manchester, Polanyi told Szilard that the research on a nuclear chain reaction ought to be done, but no money materialized (Fig. 5).

Polanyi had read *The World Set Free*. He knew Soddy, his diminished scientific reputation, and his unorthodox politics. He was one of the first to know of Szilard’s theory of the chain reaction. Whatever else was going on in that radio booth in January 1945, it is implausible that Polanyi had never connected Einstein’s theory of special relativity to any practical consequences.

## Soddy, You Shoes too Big

After the Great War, Soddy – having given up his science – lost credibility about precisely those things he was passionate about. He forfeited too much, and achieved too little, to serve as a model for scientific responsibility. His experience recalls that of other scientists who have turned to popularization and activism, including Carl Sagan, whose later career was far from valorized by his community of cosmologists. And while Lewis Mumford (1958: 12) eulogized Soddy’s “own too lonely example” and mourned awaiting “a whole generation of Soddys,” it is too hard to ask other scientists to walk even a short distance in his shoes.

Soddy’s career prior to the Great War is a more reasonable model of responsibility for contemporary scientists. Prescience aside, Soddy took a serious question seriously, grappled with the matters of the day as they related to his scientific research, engaged the public about these issues even as he was grappling with them, and envisioned a variety of “possible technical uses” of his work even when he had no idea of how, specifically, it would play out. This last characteristic is the one that distinguishes him from Polanyi, who to his credit was performing these other tasks when crammed into the radio booth.

Are scientists today capable of fulfilling such responsibilities? Science is more highly bureaucratized than it was in Soddy’s day.^7^ While they may enter research to do good, scientists find themselves mired in the formalities of funding, publishing and patenting that pre-empt this impulse. Perhaps even a relaxed version of Soddy’s responsibility resides troublingly out of reach for contemporary scientists.

But there is help. After his “glance into the abyss,” Soddy began to travel in more social scientific circles – as his relationship with Mumford attests – and actively engage in research with an economic and social scientific perspective. While such pursuits were still looked down upon by important parts of the scientific establishment, including whoever wrote Soddy’s official Nobel biography, another view of the social sciences was beginning to emerge. As Detlev Bronk, who would become the president of Johns Hopkins University, argued before the Congress in 1945 in supporting the proposal for a new National Science Foundation: “Competent social scientists should work hand-in-hand with natural scientists, so that problems may be solved as they arise, and so that many of them may not arise in the first instance” (Bronk 1975: 413).

To succeed in integrating the social and natural sciences, we have to believe, with Soddy but not Polanyi, that it is worthwhile to talk about outcomes prior to the completion of the puzzle. Soddy’s record shows, indeed, that such anticipations may be as fruitful as predictions.

## Anticipatory Governance

Anticipation of the outcomes of research, engagement with the public over research as it is being done, and integration of natural and social science are the core elements of a developing program called “anticipatory governance” (Barben et al. 2008; Guston 2008). Nurturing these capacities will create our best chance at not blundering into synthetic biology, nanotechnologies, and geoengineering as we blundered into the atomic age.

The US National Nanotechnology Initiative (NNI) has begun implementing a twenty-first century version of Bronk’s post-war directive. Through two centers for nanotechnology in society (at Arizona State University and at University of California, Santa Barbara), NNI supports studies of the governance of nanotechnology in anticipation of many if not most of its major societal ramifications. The expressed purpose of this roughly $25 million for the two centers over ten years is to support the NNI’s strategic goal of “responsible development” of nanotechnology.

Rather than attempting to predict where nanotechnologies might be in 20 or 50 years, the NNI (2007) endorsed the development of scenarios that serve as informed provocations for scientists, pedagogical tools for their students, and plausibly concrete examples for public deliberations.

Rather than informing the public of research as a *fait* *accompli*, the productive role of public engagement was on NNI’s agenda. NNI’s authorizing legislation (Public Law 108–153; section 2(b)(10)(D)) provides for “public input and outreach to be integrated into the Program by the convening of regular and ongoing public discussions, through mechanisms such as citizens’ panels, consensus conferences, and educational events, as appropriate.”

Rather than waiting until neatly packaged products emerge, the legislation also provides for “insofar as possible, integrating research on societal, ethical, and environmental concerns with nanotechnology research and development” (section 2(b)(10)(C)).^8^

Ensemble, these efforts may begin to change how scientists and engineers talk about their research and their responsibilities, as well as how they make some decisions in their laboratories.

Nevertheless, such social science and ethics research has been but tiny part of NNI’s endeavor – less than one percent of the billions that the US government invests in nanotechnology (Guston under review). Despite real efforts, neither of the two major efforts at synthetic biology in the US – the Synthetic Biology Engineering Research Center, funded by the National Science Foundation, nor the Venter Institute, performing privately funded synthetic biology research – has created robust capacities for anticipatory governance. And while beginning to take questions of governance seriously, geoengineering advocates convened a major discussion at the Asilomar conference center in California, invoking by their very location a major episode in the history of genetic engineering best remembered as an effort in autonomy that Polanyi might have appreciated.

We are thus in many ways still in 1935. Soddy (1975 [1935]: 7) believed *The Frustration of Science* to be “indicative of the growing sense of social responsibility, among some individuals of scientific merit at least, for the world the labours of their order have so largely created.” He further held that:

The public expect far more from scientific men in this respect than they have as yet contributed. Individually most of them in this field are still utterly unscientific, and quite as apt as the public themselves to regard individual thought on these subjects as socially dangerous and to be suppressed and those who have strayed from the path of “pure” science in these directions as cranks or imposters….

On the other hand, the public must not expect too much. They are apt to forget that in effect, as an entity with power of acting, they hardly exist, until *in extremis* when it is too late. The pioneer and bearer of a new evangel is always up against an inchoate mass, educable only when miserable and, when prosperous, too proud to learn….

The solution is for the public to…require that its universities and learned societies should no longer evade their responsibilities and hide under the guise of false humility as the hired servants of the world their work has made possible, but do that for which they are supported in cultured release from routine occupations, and speak the truth though the heavens fall (Soddy 1975 [1935]: 7–9).

The anticipatory governance of emerging technologies is dedicated to mediating the conflicted scientists and public in Soddy’s account, moderating the belief that such discussions are “socially dangerous,” modulating the scientists’ behavior toward general responsibility, and remediating the public’s tendency to wait until tragedy strikes before it is willing to learn.

## Prediction, or Plausibility?

As young Polanyi anticipated from his reading of *The World Set Free*, after World War II, he left chemistry and moved toward philosophy. One of his great and enduring contributions there is the concept of tacit knowledge, which is that part of science that cannot be encoded but is embodied in skill or held in intuition.

In his essay “The growth of science in society,” also published in *Minerva*, Polanyi (1967) retreats from his focus on prediction and introduces the concept of “plausibility” to settle disputes between science and pseudo-science. Plausibility explains why scientists may disregard some hypotheses, despite their leading to precise predictions: “Only plausible ideas are taken up, discussed and tested by scientists….[T]he assessment of plausibility is based on a broad exercise of intuition guided by many subtle indications, and *thus it is altogether undemonstrable. It is tacit*” (Polanyi 1967: 536; italics in the original).

Tacit assessments allow scientists to dismiss with prejudice the ideas like those, in Polanyi’s example, of Immanuel Velikovsky, who in the early 1960s hypothesized that a rogue comet born from Jupiter caused numerous events described in the Hebrew bible and then settled into orbit as the planet Venus. Velikovsky predicted, contrary to the expectations of planetary scientists, that Venus was beastly hot and held an atmosphere rich in hydrocarbons. The Mariner 2 spacecraft confirmed his predictions, yet he remained a crank and imposter to most scientists. Perhaps Polanyi felt the same way about Soddy.

Polanyi offers three “subtle indications” of plausibility that make a hypothesis or finding interesting to science: reliability, or exactitude; systematic importance to the rest of scientific knowledge; and intrinsic interest of the subject matter.

Only reliability or exactitude seems in question with Soddy’s beliefs about an atomic bomb. One is tempted to evaluate exactitude or reliability with reference to the puzzle metaphor by asking, “Are all the pieces in place?” Polanyi himself might have approached it this way, as he introduced the puzzle metaphor in “The republic of science” and returned to it in “The growth of science in society.” But the pieces fall into place for different people at different times. For Szilard, it was 1933, after Rutherford’s “moonshine” speech. For others, it was 1938 when Hahn, Frisch and Meitner identified the fission of uranium. For Polanyi, it wasn’t until the mushroom cloud manifest itself.

Having all the pieces in place, or even all of them face up, is too great a demand to place on plausibility: that is the burden of likelihood, or eventuality. Derived from the Latin for deserving of applause and thus attached to the appreciation of an audience, plausibility is a more generous concept. Pushing Polanyi’s puzzle metaphor, plausibility is more about having enough of the pieces turned up that you begin to appreciate what the complete picture might be. Enough orange and black pieces would have you thinking tiger or pumpkin.

If you are assembling bits of reality, is it not at this point that you want to start asking, “what happens if it is a tiger?”

## Back in the Booth

While the Brains Trust was wildly popular, George Orwell (1944) mocked its “phony pretense” in his *Tribune* column, holding it “a very dismal thing.” Nevertheless, he concedes that it was originally a step forward in radio programming, and that it continued to goad the reactionary, blustering “Blimps” with its agnostic, academic approach.

Each live airing of the Brains Trust was recorded automatically on a wax cylinder by a telediphone machine, a portable recording device that the BBC also used for war correspondence. The Brains Trust program on which Polanyi and Russell appeared was, according to the transcript from those wax cylinders, “Telediphoned – Monday, 8th January, 1945.”

The Question Master opened the show by doubting “whether the Brains Trust ever had quite such a constellation of scientific talent as we have round the table this week.” He introduced Russell and then Polanyi “who, like Bertrand Russell, has received to the great accolade of the scientific world the Fellowship of the Royal Society.” He introduced a medical practitioner who was also a Member of Parliament, an anonymous psychoanalyst, and a “scientific story teller.”

Toward the end of the show, after the sort of dreary questioning Orwell despised, the Question Master offered “[t]he next question…from Doctor Austin H. Birch of Newport. Does it make any practical difference whether relativity is true or not? Does it make any practical difference whether Einstein’s right or not?”

Page two of telediphone cylinder six conveys Polanyi’s recorded response:

“I’m very much convinced that in the long run the difference may be profound. It has been suggested already, recently, that with the theory of relativity, and particularly with its more general forms, which have been elaborated since Einstein’s work, we get to a conception of the world which is much closer to the medieval conception of the world than the Newtonian idea of the world was. As it were everything, all the elements of the world, arise and were derived from its structure. It’s all built in, so to speak, into the walls, or into space….”

The question posed by Dr. Birch to the “constellation of scientific talent” that January day about “any practical difference” of relativity is ambiguous on two counts: It is not clear that “practical difference” meant technological application, as Polanyi took it to mean in “The republic of science,” as opposed to, say, common meaning or importance. Neither is it clear whether relativity referred to Einstein’s special relativity or his later general relativity. What is clear, however, is that Polanyi’s response in the radio booth addresses general relativity, while his anecdote in the essay a decade and a half later addresses special relativity.

The difference is not an esoteric distinction between two theories too abstruse for ordinary minds. It is, rather, the distinction between an accurate recounting of evidence for his argument that science is unpredictable and hence ungovernable, and his inaccurate reporting of evidence that suits his argument – by a man for whom “the discovery and possession of truth [is] one of the first obligations of a good society.”

Polanyi’s reading of Wells, his scientific and political jousting with Soddy, and his encouragement of Szilard all suggest that he could not have been oblivious to the connection between special relativity and the atomic bomb. We cannot know if perhaps Polanyi had the bomb in mind on live radio in January 1945, but patriotically steered his answer away from it and toward general relativity. Yet, by 1962, there was no secret weapon to protect. He could neither be covering for the war effort, which was over, nor even for his own ignorance, which he overstates.

He was covering for the autonomy of science.

## Footnotes

1 I use the 2000 reprint of Polanyi’s original 1962 article because it is the most accessible version. Another accessible version is reprinted in Shils (1968).

2 Description from an image viewed at: http://www.turnipnet.com/whirligig/radio/brainstrust.htm.

3 Brownhill and Merricks (2002) juxtapose Soddy, Polanyi and J.D. Bernal in an informative discussion of scientific responsibility and the public. While we largely agree on Soddy and Polanyi – and while the addition of Bernal creates a broader spectrum of possibilities, especially in consideration of the more public debate between Bernal and Polanyi – I emphasize here the relationship among prediction, anticipation, and responsibility that Soddy exemplifies.

4 I am indebted to Peter Collins, historian of the Royal Society, for his efforts to locate for me a photograph of the lecture hall in Burlington House which, while dated shortly after Rutherford’s death, depicted the hall as it would have appeared during the discussion of heavy hydrogen (but for Rutherford’s own portrait).

5 In providing a brief account of the scientific humanists and Polanyi’s founding of the Society for Freedom in Science, Barber (1990: 5) highlights the “somewhat better sociological understanding of the possibilities of predicting and planning science, both in its discoveries and its social consequences.” It is precisely this understanding I am hoping to resuscitate.

6 Reviews found in Fanning (1915).

7 Contrast, for example, Weber’s (1991 [1918]) “Science as a Vocation” with Hackett’s (1990) follow-on.

8 For more on P.L. 108–153, the 21st Century Nanotechnology Research and Development Act of 2003, see Fisher and Mahajan (2006).
